# Advancing Our Understanding of the Chronically Denervated Schwann Cell: A Potential Therapeutic Target?

**DOI:** 10.3390/biom12081128

**Published:** 2022-08-17

**Authors:** Liam A. McMorrow, Adrian Kosalko, Daniel Robinson, Alberto Saiani, Adam J. Reid

**Affiliations:** 1 Blond McIndoe Laboratories, Division of Cell Matrix Biology and Regenerative Medicine, School of Biological Sciences, Faculty of Biology Medicine and Health, Manchester Academic Health Science Centre, University of Manchester, Manchester M13 9PL, UK; 2 Department of Plastic Surgery & Burns, Wythenshawe Hospital, Manchester University NHS Foundation Trust, Manchester Academic Health Science Centre, Manchester M23 9LT, UK; 3 School of Materials & Manchester Institute of Biotechnology, Faculty of Science and Engineering, University of Manchester, Manchester M13 9PL, UK

**Keywords:** peripheral nerve injury, Schwann cells, peripheral nerve regeneration, nerve injury, c-Jun, chronic Schwann cell denervation

## Abstract

Outcomes for patients following major peripheral nerve injury are extremely poor. Despite advanced microsurgical techniques, the recovery of function is limited by an inherently slow rate of axonal regeneration. In particular, a time-dependent deterioration in the ability of the distal stump to support axonal growth is a major determinant to the failure of reinnervation. Schwann cells (SC) are crucial in the orchestration of nerve regeneration; their plasticity permits the adoption of a repair phenotype following nerve injury. The repair SC modulates the initial immune response, directs myelin clearance, provides neurotrophic support and remodels the distal nerve. These functions are critical for regeneration; yet the repair phenotype is unstable in the setting of chronic denervation. This phenotypic instability accounts for the deteriorating regenerative support offered by the distal nerve stump. Over the past 10 years, our understanding of the cellular machinery behind this repair phenotype, in particular the role of c-Jun, has increased exponentially, creating opportunities for therapeutic intervention. This review will cover the activation of the repair phenotype in SC, the effects of chronic denervation on SC and current strategies to ‘hack’ these cellular pathways toward supporting more prolonged periods of neural regeneration.

## 1. Introduction

The peripheral nervous system (PNS) is hailed for its regenerative capacity, yet a proximal injury to a significant peripheral nerve, such as in the brachial plexus, brings long-term, life-changing functional and psychosocial consequences for the patient [1,2]. These are common injuries, frequently occurring in a young, working-age demographic and carrying a significant socioeconomic burden [3]. Despite a clear impetus to improve upon current outcomes, there has been little progress since the concept of prompt surgical neurorrhaphy was described by Paul of Aegina (625–690) [4] over a thousand years ago. Whilst our knowledge of nerve physiology and access to advanced microsurgical techniques should have improved outcomes, the rate of axonal regeneration, estimated at approximately 1 mm per day, is the major rate-limiting factor [5]. This slow rate of regeneration permits a switch from the acute to the chronic injury phase, whereupon additional challenges develop, including scar formation, Schwann cell (SC) failure and end-organ atrophy, such as target muscle wasting.

SC are the glia of the PNS. These remarkably plastic cells adopt a range of phenotypic changes throughout their lifetime to manage and support the complexities of axonal physiology, development and repair. In mature health, they are found either as myelinating SC, supporting and insulating single large axons, or as the Remak phenotype, engulfing multiple small diameter axons [6]. Following injury, they adopt the repair SC (rSC) phenotype and play a crucial role in myelin clearance, immune system activation, neurotrophic support and axonal guidance. Much work has focused on the role of the rSC in the acute phase; however, recent findings suggest that an instability of the rSC phenotype in the distal nerve stump over an extended period of denervation is the predominant cause of regeneration failure. 

This review will seek to summarise the cellular machinery that enables the adoption of the rSC phenotype and the deterioration of this phenotype in chronic denervation and explore whether current knowledge surrounding these changes might be targeted for therapeutic gain.

## 2. Cellular Adaptivity of SC—The Molecular Activation of the Repair Phenotype

**Figure 1:**
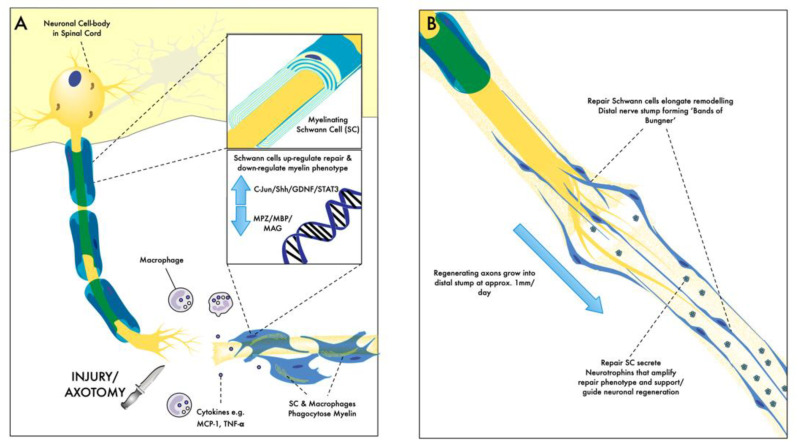
(**A**) Following axotomy, SC undergo a phenotypic switch to a repair phenotype. This switch involves the suppression of genes associated with myelination, e.g., MPZ/MBP/MAG, and the upregulation of c-Jun/Shh/GDNF/STAT3, which initiate the repair phenotype. In this state, SC initiate an immune response through the release of cytokines (e.g., TNF-α) and monocyte chemotractants (e.g., MCP-1). They activate macrophages and alongside macrophages phagocytose myelin. (**B**) The activation of c-Jun in SC initiates considerable morphological changes with extensive elongation forming the bands of Büngner, remodelling the distal stump, and bridging nerve gaps. Concurrently, they secrete neurotrophins to support and guide axonal regeneration. With chronic denervation and over time, this repair phenotype is ultimately lost.

Following axotomy, the distal axon loses connection with its cell body. Both Remak and myelinating SC undergo adaptive cellular reprogramming to adopt the rSC phenotype. In this role they execute the following programme to create the optimal environment for axonal regeneration (Figure 1):

1. The initiation of an immune response with the macrophage recruitment and activation of myelin autophagy. There is a marked downregulation of myelin related proteins, including Myelin Protein Zero (MPZ), peripheral myelin protein (PMP22), Early Growth Response 2 (EGR2, also known as krox20), Connexin-32, Myelin Basic Protein (MBP), Myelin-associated glycoprotein (MAG) and periaxin [7]. There is an upregulated production of monocyte chemotractants, Interleukin-1β (IL-1β), Tumour Necrosis Factor-α (TNF-α) and inducible Nitric Oxide Sythase (i-NOS) [8,9]. Both activated macrophages and SCs remove and phagocytose the axonal and myelin debris [10] Macrophages also play a role in converting SCs to a regenerative phenotype via secreted Interleukin-1 (IL-1), which induces SC nerve growth factor (NGF) secretion. The Macrophage secretion of Transforming Growth Factor-β (TGF-β) and Insulin-like growth factor 1 (IGF-1) encourages mitotic activity in SC, reaching a peak 3–4 days after injury [11].
2. The rSC upregulation and secretion of neurotrophic agents (glial derived neurotrophic factor (GDNF), brain-derived neurotrophic factor (BDNF), neurotrophin-3 (NT-3) and vascular endothelial growth factor (VEGF) supports neuronal survival and growth [12].
3. rSC proliferate and undergo morphological changes with significant elongation (three-fold) to form the bands of Büngner, which remodel and prepare the basal lamina for the regenerating axon [13].

It is important to state that neuronal development is a distinct biological process to mature nerve regeneration [14] and whilst there are shared pathways between the two there are also unique transcriptional pathways governing the latter, and these are discussed below.

### 2.1. c-Jun

c-Jun, a transcription factor product of the gene JUN and activated by JNK, functions as a single independent initiator of the rSC phenotype. In health, SC c-Jun levels are low [15,16] and during neural development, c-Jun is dispensable with axonal growth proceeding without c-Jun, [17]. In injury, c-Jun is essential for nerve regeneration [16]. After axonal injury, there is loss of contact between axon and SC, leading to a rapid increase in c-Jun within SC [18,19,20]. c-Jun is then suppressed when axonal contact is reinitiated, allowing a switch back to a myelinating phenotype. In SC, c-Jun controls the expression of 172 genes, including the upregulation of Shh, Olig1, BDNF, Artemin, GAP-43 and P75NTR and the suppression of myelinating genes MPZ and MBP [17]. The Sonic Hedgehog (Shh) gene is repressed by SC during development and in health but in injury is upregulated by c-Jun [17,21]. The Shh protein in turn stimulates SC c-Jun further [22] creating an autocrine signalling loop [16]. Shh antagonists suppress c-Jun [23] and thereby axonal growth, macrophage recruitment and myelinophagy [24,25]. Several other mediators are upregulated and perpetuate the rSC phenotype, including Neuregulin-1 [26,27] GDNF [28] and TGF-β [29] (reviewed in [16]).

Neuregulin-1′s role in the rSC phenotype is complex and unlike c-Jun, Neuregulin-1 also has a key role in neural development [30]. Whilst high levels of neuregulin promote the rSC phenotype in a concentration-dependent manner, lower levels support myelination [31]. Neuregulin-1 has wide-reaching effects through the activation of multiple cellular signalling pathways, including ERK1/2 in SC (reviewed in [32]).

c-Jun also initiates morphological changes whereby SC elongate to form the bands of Büngner. c-Jun supressed SC do not elongate and subsequently adopt a grossly abnormal morphology [17]. Gomez-Sanchez et al. [13] demonstrated a strong association between 2–3 fold SC elongation and elevated c-Jun levels. c-Jun activation in the setting of nerve injury is diminished with advancing age, and this correlates with a reduced capacity for nerve regeneration observed clinically in elderly populations [16].

### 2.2. STAT3

STAT3 (signal transducer and activator of transcription 3) is a transcription factor encoded by the STAT3 gene. STAT3 is activated in SC in the setting of peripheral nerve injury [33]. As with c-Jun, STAT3 is not required for neural development or myelination [34]. In STAT3 knockout models, there is decreased c-Jun and neurotrophic factor expression following injury. STAT3 improves SC survival in vitro and in vivo and the inhibition of STAT3 through JAK2 inhibitors increases the risk of apoptosis in SC [34] supporting a role for STAT3 protecting SC from death following nerve injury. Chen et al. [35] also demonstrated that STAT3 modulates SC proliferation and migration in vivo.

### 2.3. Merlin/YAP

The tumour suppressor protein Merlin [36] is also involved in the switch to rSC independent of neurodevelopmental pathways. Loss of Merlin causes the injury-dependent activation of YAP (Yes Associated Protein, a member of the hippo pathway) in SC with decreased c-Jun activation and severely impaired PNS regeneration. The removal of YAP rescues this condition and restores the regenerative capacity [36]. YAP is also involved in SC mechanotransduction suggestive that mechanical sensing may influence the rSC phenotype and stability [37].

### 2.4. Epigenetic Signalling/Chromatin Remodelling

The above-described transcription factors and signalling pathways are also subject to strict controls at an epigenic level. The post-translational modification of histones, the structural core of the nucleosome, exert a major control over chromatin accessibility. Histone modifications are diverse and may include methylation/demethylation, acetylation/deacetylation and phosphorylation/dephosphorylation. The identification and characterisation of these histone modifications and their ability to correlate with silenced or active enhancers/promotors of gene transcription has been the subject of intense research over the last 15 years.

Histone modification nomenclature follows the format of [histone name e.g., H2] + [amino acid abbreviation e.g., k] + [amino acid position e.g., 4] + [type of modification e.g., me (methylation)] + [the number of modifications e.g., 1]. The H3K4me3 modification is therefore a trimethylation of the fourth lysine on histone 3. H3K4me3 corresponds well with the areas of active transcription and is therefore frequently used to identify active promotors [38]. The marker H3K27ac is associated with active enhancers. Using chromatin immunoprecipitation techniques (chIP-seq), Hung et al. [39] demonstrated a variety of enhancers activated following nerve injury in SC, including enhancers proximal to GDNF, Shh and P75. They also demonstrated that many injury-induced enhancers have enriched binding sites for c-Jun.

H3K27me3 is a histone marker functioning as a repressor, and hence associated with closed/poised enhancers/promotors. Poised chromatin refers to that which is bivalent and marked with both active and repressor modifications, e.g., H3K4me3 and H3K27me3. This is a form of epigenic preprograming, allowing for the quick activation of transcription with the demethylation of H3K27me3, hence the term poised. Generally, poised chromatin is associated with pluripotency and most work investigating its significance has explored embryonic and developmental stem cell models. The dual marking of these genes allows them to later resolve to silent or active gene states with progressive differentiation [38]. H3K27 methylation is catalysed by Polycomb Repressive Complex 2 (PRC2) and the suppression of PRC2 has been shown to initiate of 30% of the genes typically expressed following peripheral nerve injury (e.g., Shh) [40,41]. H3K27me3 is found repressing the promotors of rSC genes preinjury (including BDNF, GDNF and Shh) [41], whereas most rSC enhancers are not poised (marked with H3K27me3) preinjury [39,40,41,42,43]. The activation of closed enhancers would therefore be dependent on c-Jun (or other transcription factors) acting as a pioneer factor. The activation of these enhancers is necessary for proper activation of rSC gene expression [42]. Therefore, the activation of rSC injury genes following insult would appear to involve: 1. the c-Jun activation of distal enhancers and 2. the demethylation of H3K27me3 to enable c-Jun-driven rSC gene expression. However, contrary to this, Duong et al. [44] recently demonstrated using knockout models that H3K27me3 demethylases are not required for the activation of the repair phenotype.

The histone deacetylases (HDACS), which, through their suppression of histone acetylation, suppress c-Jun activation, are also a subject of intense research. The HDACs are divided into four classes of which classes I (HDAC 1, 2 3 and 8) and IIa (HDAC 4, 5 7 and 9) have been explored regarding significance to SC and nerve injury. HDACs do not interact with DNA directly but through chromatin remodelling complexes, which usually also contain other remodelling enzymes e.g., demethylases. Their actions however extend well beyond simple acetyl and the deacetylation of histone proteins, as they also interact with non-histone proteins. For example, in the conversion from the rSC to the myelinating state, Brugger et al. [45] demonstrated that HDAC 2 complexes with Sox10 and H3K9 demethylases to reenable the cellular programming for remyelination. Generally, HDAC suppression enables the repair SC state [46,47] and HDAC upregulation enables the myelinating state [45]. The class IIa HDACs (despite their name) demonstrate little to no deacetylase activity due to a tyr-to-his mutation at their catalytic site [48]. Gomis-coloma et al. [49] demonstrated that in response to cAMP signalling, HDAC 4 is shuttled to the nucleus where it interacts with HDAC 3 complexed with NCoR1 to deacetylate histone 3 at the promotor of c-JUN, preventing its expression [49].

The characterisation of HDACs is further complicated by a significant degree of genetic redundancy. Whilst knockout HDAC 1 and 2 lead to impaired myelination and SC survival, this is not seen with single knockout models [50]. Even greater compensatory mechanisms are seen in the class IIa HDACS. Typically, only HDAC 4, 5 and 7 are expressed in the SC. Velasco-Aviles et al. [51] showed that a compensatory increase in HDAC 7 in response to HDAC 4 + 5 knock-out is mediated by JUN in vitro and in vivo. HDAC 4 binds to the promotor of HDAC7 and it has been postulated that the depletion of this factor then allowed binding and promotion by JUN. The suppression of HDAC 4,5 and 7 led to de novo HDAC 9 expression via the transcription factor MEF2. These compensatory mechanisms allow myelination to proceed (albeit with delay) despite the knockout of all normally expressed class IIa HDACs in SC. There is likely the involvement of other class HDACs in peripheral nerve injury which will require further elucidation. In a recent comprehensive review of the emerging HDACs and peripheral nerve injury field, Gomez-Sanchez et al. [52] analysed RNA sequencing data from Wagstaff et al. [23] and demonstrated a significant upregulation of HDAC 11 and downregulation of HDACs 6 and 8 in chronically injured nerves. There is little data investigating the effect of chronic denervation on HDACs beyond this. Despite the complexities described regarding the role of these enzymes, several authors have attempted the manipulation of their expression with a variety of pharmaceutical agents for therapeutic gain and this is discussed further in Section 4.

### 2.5. Other Pathways

The signalling pathways discussed above are not an extensive list of all rSC phenotypic modulators but are most notable due to their specificity to the activation of rSC (especially c-Jun). There are other pathways that positively regulate the rSC and neuronal regeneration but are also implicated in neurodevelopment and/or myelination. For example, Notch signalling is complex and involved both in immature and adult SC. Notch is suppressive of myelination [53] and its activation can amplify the rSC phenotype leading to improved functional regeneration [54]. Sox2, a transcription factor associated with stemness is also inhibitory of myelination and is involved in the SC nerve bridge formation when a gap between distal and proximal nerve stumps following axotomy exists. Sox2 mediates ephrin-B/ephB2 between fibroblasts and SC enabling directional organisation of SC to guide regenerating axons [55].

Lastly, the MAPK/ERK pathway has a complex and important role in neural development (along with many other cellular processes) and ERK1/2 is involved both in myelination [56] and in the rSC phenotype [57]. PNS injury initiates activation of ERK [58] but its downstream targets or involvement with c-Jun are not clear [53].

## 3. Failure of Regeneration—The Loss of the Repair Phenotype

Over the past 80 years, research has established that the prolonged denervation of an axotomized distal nerve stump instigates a progressive reduction in its capacity to support nerve regeneration. SC failure within the denervated stump has been repeatedly implicated as a prime causative agent behind this phenomenon; however, only recently (reviewed below and in Table 1) has progress been made in characterising this failed SC phenotype. 

In 1942, Holmes and Young demonstrated a link between delayed nerve repair (by three months) and diminished nerve regeneration [59]. In 1995, Fu et al. [60] explored the effects of extended muscle denervation in rats using a cross-suturing model, whereby the common peroneal nerve is cut, and after varying periods of denervation, the distal stump is reattached to the freshly cut tibial nerve proximally. Denervation reduced functional recovery secondary to a dramatic fall in the number of axons regenerating through to the muscle. Interestingly, those few axons successfully reaching muscle innervated 3–5 times more muscle fibres than the control. This suggests that prolonged denervation had not impacted the muscle fibres’ capacity for reinnervation. In a similar rat model, Vuorinen et al. [61], assessed the distal stump morphology with electron microscopy and the capacity for regeneration after periods of 3–16 months denervation. Although axons were able to regenerate even in the 16-month group, the number of axons decreased substantially with prolonged denervation. Morphologically, the deterioration of SC columns was seen in the majority, replaced by thin collagen filaments by 12 months [61]. Sulaiman and Gordon [62] showed that denervation of rat sciatic nerves up to 4 weeks had no detrimental effect on regeneration, but beyond this, there was a profound reduction in the number of axons able to regenerate through the distal stump. Jonsson et al. [63], again in a rat model, showed that chronic denervation leads to a progressive decline in SC markers in the distal stump and a lower yield of SC that can be isolated from distal stumps. Ronchi et al. [64] also examined the SC phenotype in a chronic denervation rat upper limb model and demonstrated decreased Neuregulin-1 expression with prolonged denervation.

Most recently, in 2021, Wagstaff et al. [23] provided key data to characterise the molecular machinery behind the denervated SC phenotype. In a mouse model of sciatic nerve injury, chronic denervation led to significant reductions of SC GDNF, Shh, p75-NTR and most importantly, c-Jun expression, alongside a 50% decrease in axonal growth through the denervated stump (quantified though retrograde labelling techniques). To investigate whether the restoration of c-Jun would reverse the effect of denervation, they produced a model utilising transgenic mice overexpressing c-Jun in SC only. These mice did not demonstrate c-Jun deterioration with chronic denervation, nor did they show decreased axonal growth through the distal stump with chronic denervation. These findings suggested that the restoration of c-Jun could prevent the deterioration of SC to the denervated phenotype. Further analysis with electron microscopy showed that although wild-type SC counts did decrease with chronic denervation compared to c-Jun overexpressing mice, in both groups, overall SC numbers remained considerably higher than in control nerve samples suggestive that the phenotype of, rather than the number of, SC is likely responsible for the regenerative failure through the denervated stump. Whilst all the models discussed above are rodent based, Wilcox et al. [65] explored the expression of c-Jun in chronically denervated human nerve samples, confirming that similar to rodent models, c-Jun is supressed with chronic denervation.

## 4. The Chronically Denervated SC as a Therapeutic Target

**Figure 2:**
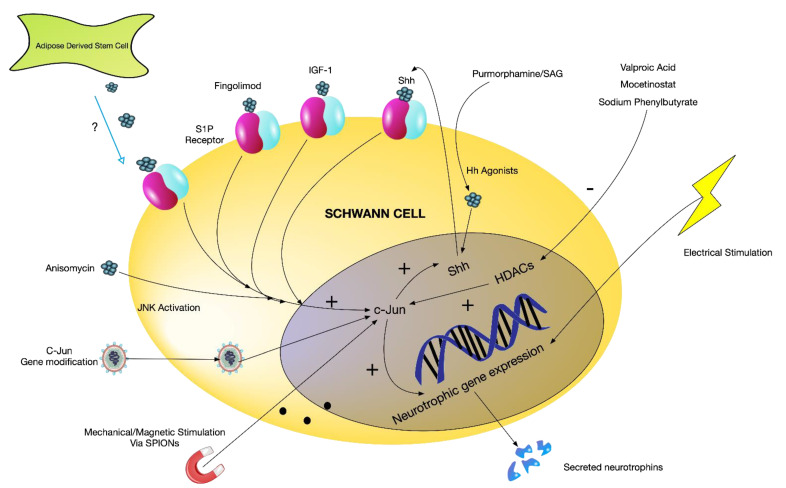
The identification of c-Jun as a key determinant of the denervated SC phenotype creates new routes for potential therapeutic intervention/investigation. The augmentation of c-Jun triggers paracrine and autocrine loops (e.g., Shh activation) that further maintain the rSC phenotype (leading to neurotrophin secretion and morphological changes that aid nerve regeneration). Several pharmaceutical agents (fingolimod/Hh agonists/Anisomycin/HDAC inhibitors) and genetic engineering strategies have been used to directly augment c-Jun and improve experimental nerve regeneration. The use of the ASC secretome, electrical and mechanical stimuli and agonists from the growth hormone axis also modulate the rSC phenotype (likely through the modulation of c-Jun) and have been explored to the same end.

Although chronic denervation may also be complicated by target muscle wasting, the formation of scar tissue and declining SC numbers in the distal stump; perhaps the single most-important feature detrimental to regeneration is the phenotypic change in SC. Our understanding of (a) the rSC phenotype, (b) the molecular signalling involved in activating and maintaining this phenotype and (c) the effect of chronic denervation on rSC instability has greatly improved over the last 10 years, creating a new paradigm and new opportunities to design therapeutic interventions around the rSC phenotype (Figure 2).

### 4.1. Pharmaceutical Intervention

Most notable in this paradigm is the role of c-Jun. We have reviewed the importance of c-Jun in the conversion of myelinating and Remak SC to rSC. Pharmacologically, Shh agonists have been utilised to increase c-Jun in vitro, activating the rSC phenotype. Purmorphamine and Smoothened agonist (SAG) (both hedgehog agonists) upregulate c-Jun expression through their activation of Shh and stimulate neurotrophic and rSC-like morphological changes. Anisomycin, an antibiotic and known JNK activator, has also been used to increase the regenerative capacity of SC in vitro through its modulation of c-Jun [22], leading to upregulated artemin and GDNF expression.

Fingolimod (currently used as an immunomodulator in multiple sclerosis) has been used to stimulate a repair phenotype in SC [70]. SC express S1P receptors (of which fingolimod is an agonist), the activation of which supresses myelination in vitro and increases in c-Jun and neurotrophic expression [70], activating the rSC phenotype.

The immunosuppressant Tacrolimus (FK506) been considered extensively as a pharmaceutical agent to improve nerve regeneration and myelination and the success of this approach has been demonstrated in vitro and in vivo [71]. Consequently, FK506 has also been studied to reverse the denervated phenotype in SC; however, although successful in acute injury, in chronic denervation there was no beneficial effect [71,72]. 

TGF-β, a cytokine produced by both macrophages and SC in the acute injury setting, has been used to reverse the chronically denervated SC phenotype in vitro with the maintenance of this phenotype when stimulated SC are transferred to in vivo conditions [71]. Interplay between TGF-β, c-Jun and SC, however, is complex, and the expression of c-Jun can control TGF-β-stimulated SC apoptosis [29].

Several pharmaceutical agents suppress members of the HDAC family (significance discussed in Section 2). The antiepileptic drug valproic acid is an inhibitor of class 1 HDACs and Wu et al. has shown that its provision both systemically [46] and locally in a peripheral nerve conduit [46] can improve peripheral nerve regeneration in a rat model through a conduit. Oct6 upregulation in the setting of nerve injury is under the control of HDAC 2, and whilst the suppression of this upregulation ultimately harms the remyelination process, the adaptive reprograming of SC to a repair phenotype is improved. Mocetinostat is a HDAC class 1 inhibitor with chief potency toward HDAC 1. Bruger et al. [44] demonstrated that the early short-term suppression of HDAC 1 and 2 with Mocetinostat supresses Oct6 upregulation, subsequently leading to potentiated c-Jun upregulation and faster overall nerve regeneration. Yadav et al. [73] suggested that the suppression of HDAC 3 in SC may modulate and supress the inflammatory state post nerve injury in association with the transcription factor NF-kappa-B p65. The authors investigated the use of the class 1 HDAC inhibitor, Sodium phenylbutyrate (PBA), in a hydrogel conduit filler for a rat nerve gap model and demonstrated improved regeneration. PBA seemed most effective at supressing HDAC 3, although its suppression of inflammatory cytokines and consequent effects on nerve regeneration may or may not directly act through HDAC3 control.

Finally, members of the growth hormone (GH) axis have been utilised to counter the effects of chronic denervation and, unlike the interventions above, which primarily target the denervated SC phenotype, GH has the added benefit of the trophic stimulation of chronically denervated muscle tissue. GH acts primarily though the stimulation of IGF-1 (insulin-like growth factor-1). In vitro GH (or IGF-1) increases SC and neuronal survival (reviewed in [74]). Improved functional recovery has been seen through the provision of GH in a rat model of chronic denervation, although it is unclear, the mechanism of these benefits and changes in rSC markers was not fully examined [75]. Hanwright et al. [76] utilised a hydrogel, releasing IGF-1 in a rat model of chronic nerve degeneration. This intervention maintained SC proliferation in chronic denervation and improved the functional outcomes of nerve regeneration [76]. The interplay between GH and c-Jun activation in SC has not been established, although in other models IGF-1 is known to stimulate c-Jun [77]. A clinical human trial investigating the long-acting GH analogue Tesamorelin in peripheral nerve injury is underway [78].

### 4.2. Genetic Engineering

Huang et al. [79] utilised a lentiviral c-Jun-IRES-eGFP vector to transfect SC in vitro to over-express c-Jun. Transfected SC demonstrated the increased expression of neurotrophins (BDNF, GDNF, LIF, NGF and NT-3 and in a co-culture system, dorsal root ganglion cultures demonstrated significantly greater neurite outgrowth. As discussed previously, c-Jun is known to inhibit myelination, a state crucial for the repair phenotype but a state that must be reversed after initial regeneration to allow remyelination. There is no long-term published experiment looking at the effect of c-Jun expressing SC and later myelination in vivo.

### 4.3. Cellular Therapy

Surprisingly, whilst numerous studies have demonstrated benefits of cellular therapies on acute nerve regeneration, there are few data examining cellular therapy addressing the chronic denervation of SC. Elsayed et al. [80] demonstrated that adipose-derived stem cells (ASC) in co-culture with SC significantly increase c-Jun expression, a mechanism which must act through the ASC secretome, but this has not been examined in chronically denervated SC.

Walsh et al. [81] found that in vivo regeneration through a chronically denervated stump could be improved through the administration of skin-derived precursor cells (SKP). SKP-treated denervated distal stumps demonstrated superior muscle reinnervation, axon counts and compound muscle action potentials.

### 4.4. Other Novel Interventions

As discussed earlier, the suppression of YAP, a member of the hippo pathway, is important in the adoption of a repair phenotype following injury. YAP is also involved in SC mechanotransduction. Harnessing the potential influence of mechanical stimuli, Liu et al. [82] investigated whether magnetic stimuli (following the SC uptake of superparamagnetic iron oxide nanoparticles) could affect the adoption of the rSC phenotype and demonstrated increased c-Jun, stat3, neurotrophic and typical repair SC morphological features with the application of magnetic stimuli.

Electrical stimulation may represent another potentially promising area of future research. Multiple authors (reviewed in [83]) have demonstrated the capacity for electrical stimulation to increase neurotrophic secretion by SC (and independently of SC; axonal growth). In vivo, the improvement in nerve regeneration seen from short-term electrical stimulation does not seem to be diminished in chronically denervated nerve stumps [84]. The exact mechanisms of this and whether this is/is not reliant on SC changes or rSC activation is yet to be elicited.

## 5. Conclusions and Outlook

Sadly, for patients with a significant nerve injury, chronic denervation is an inevitable fact. Reduced reinnervation secondary to chronic denervation is seen after 4 weeks and the quality of functional recovery falls quickly beyond this timepoint. Assuming a nerve regeneration speed of 1 mm/day, this would imply that any nerve injury more than 2.8 cm proximal to its target organ faces suboptimal recovery due to the deleterious effect of chronic denervation.

In Section 1, we questioned whether there were advances in our understanding of the chronically denervated SC that could create new therapeutic targets to which we could direct new research hypotheses. Marrying the advances described across Section 1 (the rSC phenotype) and 2 (the chronically denervated SC) it becomes evident that poor nerve recovery in the face of a prolonged regenerative distance is predominantly due to a failure of SC to maintain the rSC phenotype. We have reviewed and collated abundant evidence that the repair phenotype in SC is not a regression into an immature, neurodevelopmental state but a unique state dedicated to repair, pre-programmed at both a genomic and epigenomic level with dedicated signalling (e.g., Shh) and does not function within SC at other timepoints. This paradigm, solidified only within the last 5 years, has strengthened current attempts to potentiate the rSC phenotype in nerve repair in vitro and in vivo. In Section 3, we reviewed the potential pharmaceutical, cellular, electrical, and mechanical interventions that have, to date, demonstrated influence over the molecular machinery behind rSC. Although seemingly far away from impacting how we manage these injuries clinically, several of the potential pharmaceutical interventions reviewed are already in widespread use for other purposes (e.g., valproic acid) and through a new understanding of the rSC molecular machinery, a meaningful clinical intervention may be closer than thought. The reader will note, however, that few of the papers reviewed attempted to address or include models beyond acute regeneration toward the rescue of the chronically denervated state. Even reviewing the studies presented in Table 1, one will note that from 1942–2020, research made headway into characterising the link between chronic denervation and poor recovery but without any meaningful strategy to correct this. We can therefore answer the question posed in the title and introduction of this review with the conclusion that chronically denervated SC not only ‘can’ be a therapeutic target but ‘must’ become the chief target of any meaningful intervention to improve nerve regeneration. Knowledge regarding the molecular biology of SC and the phenotypic deterioration in denervation is such that numerous experimental hypotheses could be applied to existing models of chronic denervation (e.g., cross-suture models) but are yet to occur. Undoubtedly, the further characterisation of the behaviour of HDAC and other chromatin-remodelling enzymes will also enable new therapeutic targets, yet for now, there are numerous research questions, including most of the strategies presented in Section 3, which need to be directed toward current models of chronic denervation, a field that will remain relatively neglected until then.

## Figures and Tables

**Table 1:** Summary of key papers exploring the effects of chronic denervation on peripheral nerve regeneration over the last 80 years.

Reference	Model	Findings
Holmes W and Young 1942 [59]	Rabbit, delayed tibial/peroneal cross suture model. Histological analysis with denervation periods up to 17 months.	Reduced advancement of regenerating axons through the distal stump with increasing denervation periods.
Sunderland and Bradley, 1950 [66]	Common Brushtail Possum (Trichosurus vulpecula). Denervation of ulnar and median nerves for varying periods up to 485 days. Histological observation.	Prolonged denervation causes progressive peripheral nerve atrophy in the form of endoneurial tube shrinkage.
Fu and Gordon 1995 [60]	Rat, delayed tibial/peroneal cross suture model. Electrophysiological and histological analysis with denervation up to one year.	Prolonged denervation decreases regenerating axon count and resultant muscle force.
Vuoronin et al., 1995 [61]	Rat, delayed tibial/peroneal cross suture model. Denervation between 3–16 months. Histological and ultrastructural analysis.	Denervation results in loss of SC columns with majority replaced by fibroblasts and collagen fibrils by one year. Prolonged denervation leads to decreased axonal regeneration.
Terenghi et al., 1998 [67]	Human biopsies of peripheral nerve at various lengths of denervation (8–53 months). Histological analysis with light and electron microscopy.	Endoneurial tube shrinkage and collagen deposition with extended denervation. All axons regenerating through denervated specimens co-localize with S100+ SC. SC remain in the distal stump for up to 53 months arranged in bands of Büngner.
Sulaiman and Gordon, 2000 [62]	Rat, delayed tibial/peroneal cross suture model. Denervation between 0–24 weeks. Outcomes; Fluorogold motor neuron back labelling, electron microscopy and histological analysis.	Few axons regenerate when denervation goes beyond 4 weeks. Although fibres are larger with thicker myelin sheaths than control.
Hoke et al., 2002 [68]	Rat, delayed tibial/peroneal cross suture model. 1- and 6-month denervation groups. mRNA and protein analysis for GDNF.	Reconnection of the distal stump led to significantly increased GDNF expression in the 1-month group but not in the 6 month group, suggesting either a reduced capacity for SC to respond to renervation or a reduced number of GDNF producing SC.
Jonsson et al., 2013 [63]	Rat model of sciatic nerve injury with delayed allograft repair (from donor rat) at 1/3/6 months. Outcomes: Retrograde axonal tracing (fluoro-ruby), SC extraction for in vitro co-culture and rT-PCR. Gastrocnemius muscle weights.	Prolonged denervation reduces S100 expression in the distal stump and reduced numbers of SC are present. Denervated SC retain capacity for proliferation in vitro and improve neurite outgrowth in a co-culture model. Regeneration through denervated stumps leads to reduced muscle weight, fibre size and axon count.
Ronchi et al., 2017 [64]	Rat, delayed median/ulnar cross suture model. Control, 3- and 6-month distal stump denervation groups. Outcomes: rT-PCR of distal stumps for neuroregulin-1, MBP, S100, p75 and GFAP. Grip strength assessment and electron microscopy for ultrastructural analysis.	Denervation of 3 or 6 months completely prevented functional recovery and smaller axon numbers and density were also seen in denervated groups. NRG1, MBP and GFAP were downregulated in denervated stumps. P75 was upregulated across all groups.
Gordon et al., 2019 [69]	Rat model of denervation with sciatic nerve injury and denervation for 7 days, 7 and 17 weeks. SC extracted from denervated nerves and assess in an in vitro co-culture model.	Fewer SC were able to be isolated from denervated stumps, but denervation did not affect SC ability to myelinate DRG neurite in vitro. Proliferative capacity of SC was reduced 30% in the 17-week group.
Wilcox et al., 2020 [65]	Human healthy and denervated nerve samples. rT-PCR and immunohistochemistry to assess SC c-Jun and P75-NTR	Chronic denervation in human peripheral nerve mirrors rodent models with decreased c-Jun and p75-NTR expression.
Wagstaff et al., 2021 [23]	Mouse sciatic nerve model of chronic denervation with 1- and 10-week groups. IHC, retrograde tracing, mRNA and protein analysis outcomes.	Chronic denervation over 10 weeks leads to supressed c-Jun expression and reduced axonal regeneration. Restoration of c-Jun removes the denervated SC phenotype. c-Jun activation is linked to Shh activation and the provision of Shh agonists can restore c-Jun expression.

## Data Availability

Not Applicable.
